# Delirium after coronary artery bypass grafting with cardiopulmonary bypass surgery: The value of cerebral autoregulation

**DOI:** 10.1177/02676591251370076

**Published:** 2025-08-17

**Authors:** Greta Kasputytė, Birutė Kumpaitienė, Milda Švagždienė, Judita Andrejaitiene, Edmundas Širvinskas, Yasin Hamarat, Edvinas Chaleckas, Vilma Putnynaitė, Laimonas Bartusis, Rolandas Žakelis, Vytautas Petkus, Arminas Ragauskas, Tadas Lenkutis, Rimantas Benetis

**Affiliations:** 1 Institute of Cardiology, Medical Academy, 230647 Lithuanian University of Health Sciences, Kaunas, Lithuania; 2 Department of Disaster Medicine, Medical Academy, Faculty of Medicine, 230647 Lithuanian University of Health Sciences, Kaunas, Lithuania; 3 Department of Cardiac, Thoracic and Vascular Surgery, Medical Academy, Faculty of Medicine, 230647 Lithuanian University of Health Sciences, Kaunas, Lithuania; 4 Health Telematics Science Institute, Kaunas University of Technology, Kaunas, Lithuania; 5 Department of Anesthesiology, Medical Academy, Faculty of Medicine, 230647 Lithuanian University of Health Sciences, Kaunas, Lithuania

**Keywords:** postoperative delirium, cerebral autoregulation, cardiac surgery, cardiopulmonary bypass, neurological complications

## Abstract

**Introduction:**

Postoperative delirium affects up to 60% of cardiac surgical patients. No reliable gold standard method exists for preventing delirium after cardiac surgery. An example of patient-personal monitoring is cerebral autoregulation (CA). This study aims to highlight the personal monitoring of patients’ cerebral autoregulation and to determine its relationship with postoperative delirium. Additionally, it seeks to test the hypothesis that the duration of CA impairment influences the onset of postoperative delirium.

**Methods:**

A prospective study was conducted in 2021–2023. After approval of the Ethics Committee and with the patient’s written consent, 104 adult patients undergoing elective coronary artery bypass graft (CABG) with cardiopulmonary bypass (CPB) surgery were enrolled. To diagnose possible delirium, all patients underwent a Confusion Assessment Method for the Intensive Care Unit (CAM–ICU) test. CA monitoring using transcranial Doppler was performed. CA status index – Mx was recorded.

**Results:**

Our study found that 12.5% of patients were diagnosed with delirium after on-pump CABG surgery. The total duration of cerebral autoregulation impairment (TCAI) was longer in the delirium group, 4783.0 seconds versus 4204.5 seconds (*p* = .047), with a cut-off at 4380 s. Longer cardiopulmonary bypass (CPB) leads to prolonged CA impairment (*p* < .001). The mean arterial pressure (MAP) during CPB was 69.67 mmHg in the non-delirium group and 74.91 mmHg in the delirium group (*p* = .001), with a cutoff at 73.669 mmHg.

**Conclusions:**

CA impairment is crucial for delirium development after cardiac surgery. The duration of the TCAI event increases the risk of delirium.

## Introduction

Delirium in cardiac surgery is a particularly long-standing problem, with ongoing efforts to discover new recognition or management methods to reduce the frequency of this complication and improve patient outcomes. According to the Diagnostic and Statistical Manual of the American Psychiatric Association classification (DSM-5), delirium is characterised as a disorder of attention and perception, where its development can be very rapid, and the course may fluctuate and vary.^1^ An exceptionally high frequency of delirium is observed after cardiac surgeries. Although cardiac surgical, anaesthetic, and cardiopulmonary bypass techniques are very sophisticated today, the incidence of postoperative delirium among surgical patients is up to 60%.^2^

Currently, there is no reliable, optimal, and routine monitoring method in clinical practice to prevent postoperative delirium after cardiac surgery. Cerebral autoregulation (CA) is an example of patient-specific monitoring that can be used during cardiac surgery to accurately and individually prevent this complication postoperatively.

This study aims to highlight the personal monitoring of patients’ cerebral autoregulation and to determine its relationship with postoperative delirium. Additionally, it seeks to test the hypothesis that the duration of cerebral autoregulation impairment affects the development of postoperative delirium.

## Materials and methods

This study received approval from the Kaunas Regional Biomedical Research Ethics Committee (No. P1-BE-2-64/2021, dated 15 December 2021). Participants provided written informed consent in line with the Declaration of Helsinki. We enrolled 104 patients undergoing elective coronary artery bypass graft (CABG) surgery with cardiopulmonary bypass (CPB) at the Hospital of Lithuanian University of Health Sciences Kaunas Clinics. Inclusion criteria were elective CABG with CPB, no history of neurocognitive disease, and carotid artery atherosclerosis ≤50%. Patients with neurological disorders and uncontrolled diabetes (HbA1c ≥ 7%) were excluded. All patients underwent standard surgical procedures under standard anaesthetic protocols. Premedication included 1–2.5 mg of lorazepam and half of the patient’s daily dose of metoprolol. General anaesthesia was induced with 80 % oxygen, fentanyl (1–2 μg/kg), propofol (1.5 mg/kg), and rocuronium (0.6 mg/kg). Sevoflurane was maintained at anaesthetic levels, as indicated by a Bispectral Index (BIS) of 40–60, with lung ventilation using 50% oxygen and fentanyl (10–12 μg/kg) for analgesia and muscle relaxants as needed. A middle sternotomy was performed, and a cold St Thomas cardioplegic solution was employed for myocardial protection. The CPB circuit was primed with Ringer’s acetate and heparin, with an activated clotting time (ACT) of ≥400 seconds. Pump flow was maintained at 2.2–2.4 L/min/m^2^, and CPB was conducted at 35–36°C.

Postoperative delirium was assessed using the Confusion Assessment Method for the Intensive Care Unit (CAM-ICU). The test was evaluated while patients were in the ICU, every 6–8 h or more frequently as needed, in the event of changes in sedation or pain management, variations in the patient’s general condition, or cases of suspected delirium.

Cerebral autoregulation was monitored using a special head frame with a transcranial Doppler (TCD) system, which measured real-time blood flow velocities in the middle cerebral artery (MCA). Cerebral autoregulation status (mean flow index, Mx) was calculated using Intensive Care Brain Monitoring System (ICM+) software (Cambridge, U.K.). Signals were filtered with a 0.1 Hz cut-off frequency lowpass Butterworth filter. The Pearson correlation was calculated in a 30-s window due to the pulse length. A threshold of Mx < 0.4 indicated intact cerebral autoregulation, whereas a value greater than 0.4 indicated CA impairment.^3,4^ The analysis calculated the duration of cerebral autoregulation impairment events to evaluate their impact on postoperative delirium.

Statistical analysis was performed using IBM SPSS version 29.0. Normality was assessed with the Kolmogorov-Smirnov and Shapiro-Wilk tests. Qualitative variables were compared using the Pearson chi-square test. In contrast, the Mann-Whitney U test was used for non-normally distributed quantitative data, which was presented as medians with minimum and maximum values. Youden’s index was used to select the optimal predicted probability cut-off. Linear regression analysis was performed to determine correlations. A *p*-value of less than .05 was considered statistically significant.

The study protocol was registered with the ClinicalTrials.gov Protocol Registration and Results System (Identifier: NCT04943458) on June 29, 2021.

## Results

**Figure 1.**
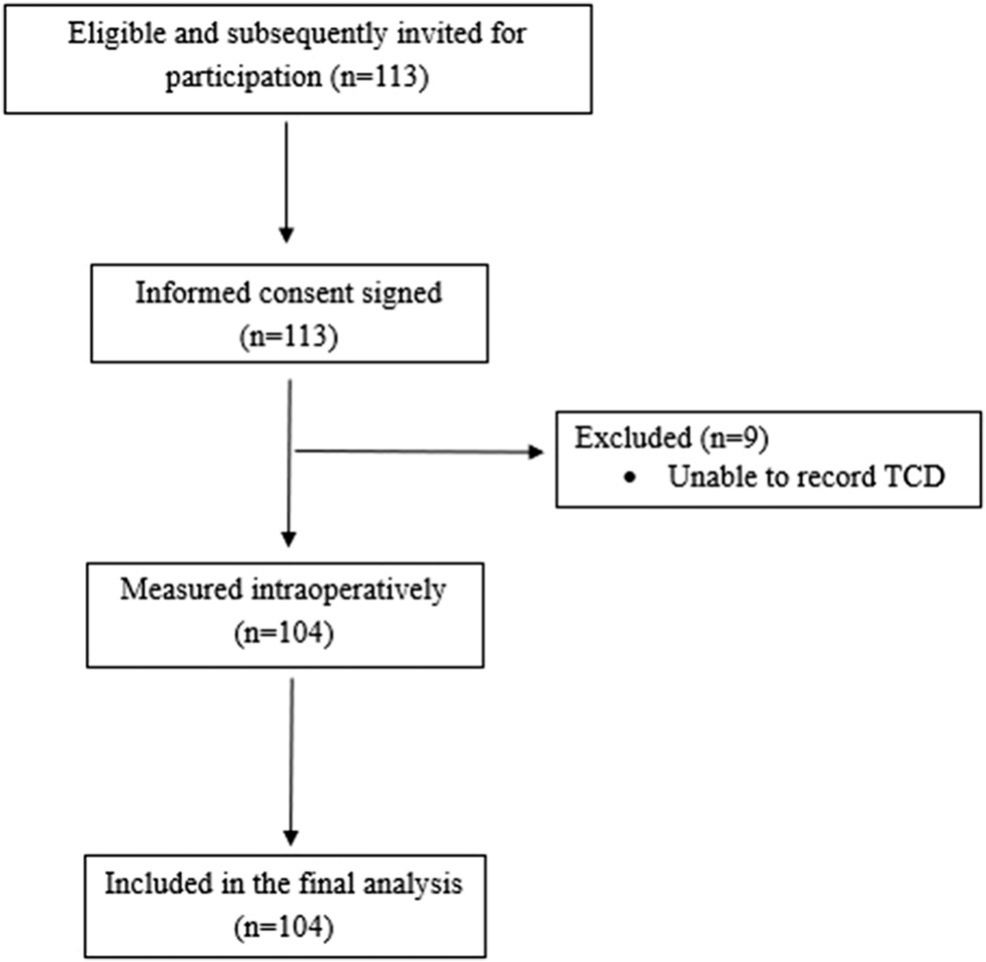
Flow chart of the study.

One hundred and four patients were enrolled in the study (Figure 1).

According to the CAM–ICU test results, patients were divided in two groups: patients without delirium after surgery were included in the first (no-delirium) group, and patients with delirium after surgery were included in the second (delirium) group.

In our study, 12.5% of patients were diagnosed with delirium following CABG on-pump surgery. Demographic and perioperative characteristics are shown in Table 1.

**Table 1.** Patient’s demographic and perioperative data according to CAM – ICU test results.

	No-delirium (*n* = 91)	Delirium (*n* = 13)	*p*-value
Sex			
Male (*n*, %)	69 (75.8)	9 (69.2)	.608
Female (*n*, %)	22 (24.2)	4 (30.8)	
Age (years)	66 (51–85)	69 (52–82)	.727
BMI (kg/m^2^)	27.59 (21–43)	27.78 (24–38)	.301
Years of education	12 (7–25)	11 (4–16)	.120
Ejection fraction (%)	52 (35–60)	50 (30–60)	.190
EuroScore (%)	1.72 (1–32)	2.44 (1–7)	.077
Diabetes mellitus (*n*, %)	13 (14.8)	0 (0)	.138
Smoking (*n*, %)	23 (26.1)	4 (30.8)	.725
AF before surgery (*n*, %)	4 (4.5)	1 (7.7)	.625
AF after surgery (*n*, %)	22 (25.6)	3 (23.1)	.846
AMI (*n*, %)	9 (10.2)	7 (53.8)	.001*
Dyslipidemia (*n*, %)	84 (95.5)	12 (92.3)	.625
Pump flow (l/min/m^2^)	4.68 (4–6)	4.6 (4–6)	.919
Norepinephrine during CPB (mg)	0.35 (0–3)	0.23 (0–1)	.222
Peripheral temperature (^°^C)	34.9 (34–36)	34.8 (33–36)	.422
Nasopharyngeal temperature (^°^C)	34.7 (33–36)	34.6 (33–36)	.396
Sodium level during CPB (mmol/l)	135 (124–140)	135 (124–137)	.890
Duration of surgery (min)	180 (140–300)	190 (180–330)	.136
Duration of CPB (min)	83 (30–130)	95 (74–166)	.036*
Duration of aortic cross-clamp (min)	40 (23–116)	47 (37–76)	.021*
MAP during CPB (mmHg)	69.67 (56.54–116.59)	74.81 (69.4–79.95)	.001*
MAP during surgery (mmHg)	79.49 (62.15–93.35)	80.9 (75.85–90.13)	.261
Duration of SLCAI (sec)	734.4 (30–3536)	384 (200–1466)	.290
Duration of TCAI (sec)	4204.5 (860–7656)	4783 (3784–9573)	.047*

Data are presented as the median (min, max) or proportion, as appropriate. The Chi-square test determined the difference in frequency distribution between groups. The Mann-Whitney U test was performed for nonparametric statistics. *Results are statistically significant. BMI: body mass index; AF: atrial fibrillation; AMI: acute myocardial infarction (30 days); MAP: mean arterial pressure; CPB: cardiopulmonary bypass; SLCAI: single longest cerebral autoregulation impairment; TCAI: total cerebral autoregulation impairment.

**Figure 2.**
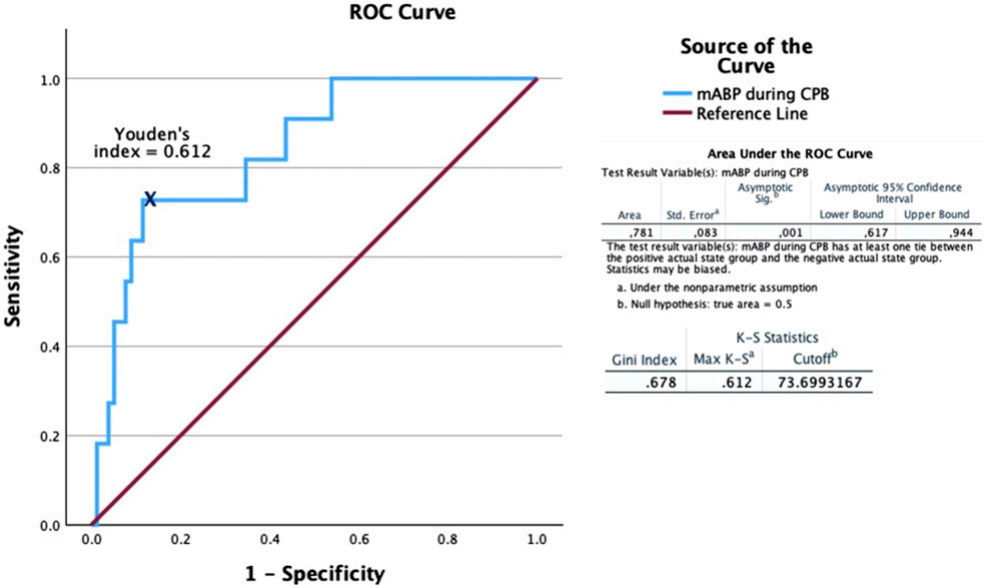
Diagnostic specificity and sensitivity of MAP during CPB in diagnosing delirium. mABP, MAP: mean arterial pressure; CPB: cardiopulmonary bypass.

After performing the ROC analysis (AUC 0.781, *p* = .001), the individual Youden’s index (0.612) was calculated when delirium develops at mean arterial pressure (MAP) 73.669 mmHg during CPB, with sensitivity (Se) at 66.7% and specificity (Sp) at 89.2% (Figure 2).

**Figure 3.**
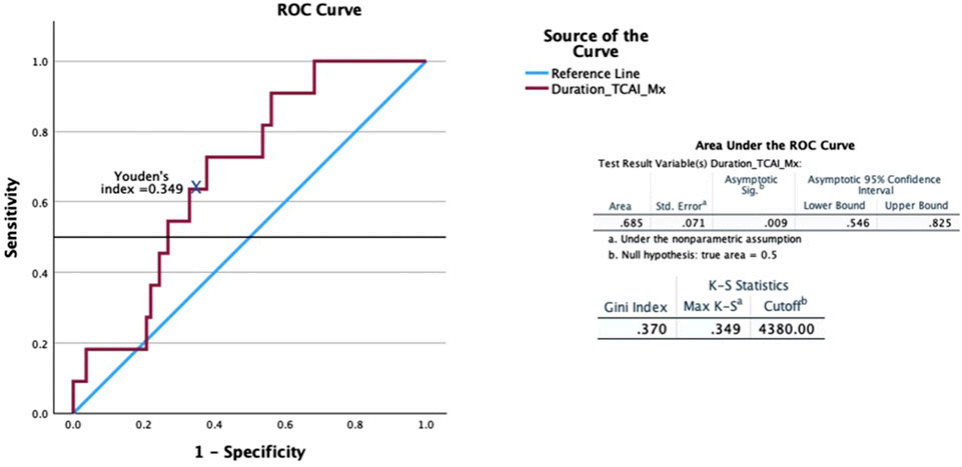
Diagnostic specificity and sensitivity of TCAI duration during CPB in diagnosing delirium. TCAI: total cerebral autoregulation impairment; CPB: cardiopulmonary bypass.

When calculating the total duration of critical CA impairment points (AUC 0.685, *p* = .009) affecting the development of delirium, the individual Youden’s index is 0.349, with a cut-off of 4380 s, a sensitivity (Se) of 72.7%, and a specificity (Sp) of 62.2% (Figure 3).

**Figure 4.**
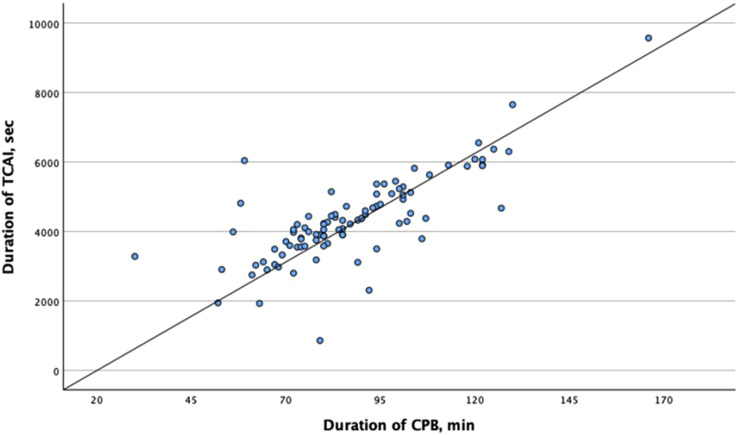
Analysis of the relationship between the duration of CPB and TCAI. TCAI: total cerebral autoregulation impairment; CPB: cardiopulmonary bypass.

Our study examined the relationship between CPB duration and TCAI event duration and revealed a moderate positive correlation, r (Pearson) = 0.778, *p* < .001 (Figure 4).

## Discussion

The incidence of postoperative delirium depends on whether we know how to diagnose it. Rudolph et al.'s study reports that only 3% of postoperative delirium is detected during routine clinical assessments. However, if we administer standardized questionnaires to the ICU patients, the incidence of postoperative delirium increases up to 53%. After CABG surgery, the frequency of delirium is reported to be relatively high, ranging from 37% to 58%, according to the same study.^5^ A meta-analysis published in 2021, encompassing 14 studies with a sample size of 13.286, found that postoperative delirium occurred at rates ranging from 4.1% to 54.9%.^2^ In our study, the CAM-ICU test was used to detect delirium, and the data from our clinic aligns with the reported frequency of delirium in the meta-analysis.

Many risk factors can influence the development of delirium after cardiac surgery: preoperative cognitive dysfunction or other neurological disorders, older age, lower education level, smoking, poorly controlled diabetes, arterial hypertension, preoperative myocardial infarction, higher European System for Cardiac Operative Risk Evaluation score (EUROSCORE), postoperative arrhythmia, longer duration of stay in the Intensive Care Unit and many others. Our study also compared the risk factors in two groups: with and without delirium. Results revealed that risk factors, such as acute myocardial infarction (30 days), duration of CPB, and aortic cross-clamp, were higher in the delirium group. These are the most common risk factors identified in other studies and included in systematic meta-analyses.^6–8^

The biggest problem in clinical practice is that most identified risk factors are irreversible and vary from patient to patient. Modern medicine focuses on individual patient monitoring, so the management of delirium in cardiac surgery patients should not be an exception. To ensure this, most cardiac surgery centers routinely use near-infrared spectroscopy (NIRS) to reduce the incidence of this complication. However, a 2017 systematic review of 1466 patients across 10 randomized controlled studies found that NIRS-based algorithms did not offer any clinical benefit.^9^ The lack of evidence led to the 2019 guidelines on cardiopulmonary bypass in adult cardiac surgery recommending near-infrared spectroscopy as a class 2B recommendation only.^10^ Other methods for individual monitoring are not recommended according to the guidelines.

Consequently, further research is needed to gain a more comprehensive understanding of the roles played by various neuromonitoring techniques and the neurological complications that may arise, such as delirium following cardiac surgery. Impaired cerebral autoregulation is important, exhibits variability among patients, and is primarily a risk factor that can be managed during anaesthesia administration.^11^ In our study, CA impairment continued for almost the entire duration of CPB. Most authors provide derived values for CA parameters, arterial blood pressure, and time, but do not report the total duration of CA impairment.^3,12^ According to research, short–term disturbances in cerebral autoregulation typically occur, but due to the brain’s ability to adapt to these changes, and do not exhibit clinical symptoms. Long–term cerebral autoregulation disorders may present as neurological complications following cardiac surgery, making it crucial to identify the total duration of cerebral autoregulation impairment. We identified only one publication that calculated the TCAI during CPB. Kumpaitiene reported that the mean duration of total CA impairment was 19.8 min, with a minimum duration of 1.4 min and a maximum of 49.9 min during cardiac surgery. When comparing the two groups, those with postoperative cognitive dysfunction (POCD) and those without POCD, the total duration in the POCD group was 24.97 min. At the same time, the duration in the other group was 17.03 min.^13^ In our study, the TCAI duration was longer, although the sample size was also larger. Therefore, we believe that the duration of TCAI is important for the development of postoperative neurological complications. However, more research is needed.

CA disorders usually develop during CPB, when the blood flow becomes non-pulsatile. Nuttal’s study found cerebral blood flow was altered during CPB in all cardiac surgery patients compared to their preoperative levels.^14^ Other studies have investigated the effects of non-pulsatile blood flow on postoperative outcomes. O’Neil M. et al. examined how microvascular flow responds to both pulsatile and non-pulsatile flow during surgery. They found that after CPB, microcirculation remained altered for 24 h, which could impact postoperative damage to vital organs.^15^ Even short-term non-pulsatile flow can influence cerebral blood flow.^16^ Our study confirms the moderate positive relationship between CA impairment and CPB. Previous investigations support these findings that CA impairment is associated with the duration of cardiopulmonary bypass.^12,17^

The duration of CPB is important for CA impairment and postoperative delirium, as well as arterial blood pressure during CPB. According to the 2024 Guidelines on cardiopulmonary bypass in adult cardiac surgery, it is recommended that the MAP during CPB should be maintained between 50 and 80 mmHg.^18^ During the study, we followed these recommended MAP limits during CPB. However, we observed that the MAP during CPB was higher in the delirium group than in the no-delirium group. Therefore, these broad MAP limits cannot be applied universally to all patients. Most CABG patients have had a diagnosis of arterial hypertension for many years. Consequently, their CA limits are shifted to the right, making it impossible to follow the rule that CA ensures cerebral blood flow within a MAP of 50–150 mmHg. According to previous guidelines, targeting the MAP during CPB within the limits of individualized CA data should be considered. Brown C.H. and colleagues compared delirium rates between standard and autoregulation-targeted groups during CPB. Delirium occurred in 53% of the standard group versus 38% in the CA-targeted group (*p* = .04), with mean arterial pressures of 71.3 mmHg for the standard group and 73.9 mmHg for the CA-targeted group.^19^ Daijiro Hori et al. determined the optimal MAP value based on autoregulation limits using transcranial Doppler (TCD). They found that the optimal MAP during cardiopulmonary bypass (CPB) was 78 ± 12 mmHg.^3^ In the Hogue et al. randomized controlled trial, delirium occurred in 8.2% of CA-based MAP target patients versus 14.9% in the standard group.^20^ Our data differs slightly from the studies described. Although the established MAP limits vary across available studies, they still support the hypothesis that the MAP range of 50 to 80 mmHg during CPB, as stated in the 2024 Guidelines on Cardiopulmonary Bypass in Adult Cardiac Surgery, is too broad and cannot be universally applied to all patients during cardiac surgery. Identifying an appropriate MAP to ensure cerebral perfusion and minimize postoperative neurological complications is challenging; therefore, further studies are required.

Our study had some limitations. It was a single–centre study with a relatively small sample size. Therefore, the results must be interpreted cautiously and confirmed by more extensive multicentre studies. Additionally, CA was monitored only in the operating room; however, the entire perioperative period, including the intensive care unit, is important for CA changes and postoperative neurological outcomes. In the future, we propose to perform CA monitoring and real–time correction of factors influencing CA impairment. Despite these limitations, the importance of this topic cannot be overstated, and we strongly believe it could be the focus of further research.

## Conclusions

Our finding supports that cerebral autoregulation impairment during CABG with CPB is an important patient-specific factor in developing delirium after cardiac surgery. The total duration of cerebral autoregulation increases the risk of postoperative delirium. To reduce it, cerebral autoregulation should be routinely measured alongside standard monitoring. This provides patient-specific data during anaesthesia, and adjustments based on this information may lower the risk of postoperative delirium during cardiac surgery.

## Data Availability

The data presented in this study are available upon request from the corresponding author.*
